# Contraceptive Use among Women with Chronic Medical Conditions and Factors Associated with Its Non-Use in Malaysia

**DOI:** 10.5539/gjhs.v4n5p91

**Published:** 2012-08-01

**Authors:** Rosliza Abdul Manaf, Irmi Zarina Ismail, Latiffah A. Latiff

**Affiliations:** 1 Department of Community Health, Faculty of Medicine & Health Sciences, University Putra Malaysia; 2 Department of Family Medicine, Faculty of Medicine & Health Sciences, University Putra Malaysia

**Keywords:** contraceptive use, chronic medical conditions, Malaysia, family planning, preconception care

## Abstract

**Introduction:**

Women with chronic medical conditions are at higher risk of adverse pregnancy outcomes, which may be minimized through optimal preconception care and appropriate contraceptive use. This study aimed to describe contraceptive use among women with chronic medical conditions and factors associated with its non-use.

**Methods:**

This study used cross-sectional data from a family planning survey among women with chronic medical conditions conducted in three health facilities in a southern state of Malaysia. A total of 450 married women in reproductive age (18-50 year) with intact uterus, and do not plan to conceive were analysed for contraceptive use. Both univariate and multivariate analysis was conducted to identify factors associated with contraceptive non-use among the study participants.

**Results:**

A total of 312 (69.3%) of the study participants did not use contraceptive. Contraceptive non-use was highest among the diabetics (71.2%), connective tissue disease patients (68.6%) and hypertensive patients (65.3%). Only 26.3% of women with heart disease did not use contraceptive. In the multivariate analysis, contraceptive non-use was significantly more common among women who received their medical treatment in the health clinics as compared to those who received treatment in the hospital (adjusted odds ratio [OR]=1.75, 95% confidence interval [CI]: 1.09, 2.79), being in older age group of 41-50 year (adjusted OR=2.31, 95% CI: 1.19, 4.48), having children (adjusted OR=4.57, 95% CI: 1.66, 12.57) and having lower education (adjusted OR=2.87, 95% CI: 1.43, 5.77).

**Conclusion:**

About two-third of women with chronic medical conditions who needed contraceptive did not use them despite the higher risk of pregnancy related complications. The high unmet need warrant an effective health promotion programme to encourage the uptake of contraceptives especially targeting women of older age group, low education and those who received their medical treatment at health clinics.

## 1. Introduction

Each pregnancy and childbirth carry a health risk for women, and the risk is magnified in women with pre-existing chronic medical conditions. Many chronic medical conditions have been affecting women in reproductive age group. However, the common types which were proven to cause maternal and fetal morbidities are hypertension, diabetes, connective tissue diseases and heart diseases. Pregnant women with hypertension and heart disease have a higher risk of developing congestive heart failure, arrhythmia, preterm delivery, intrauterine growth retardation and reduced birth weight (Cunningham et al., 2005; Domènecha & Gatzoulisb, 2006). Diabetes increases the risk of caesarean section, reduced birth weight, preterm delivery, neonatal hypoglycaemia, congenital anomalies and perinatal mortality (Evers, De Valk, & Visser, 2004; Macintosh et al., 2006; Rosenberg et al., 2005). Due to the high risk associated with pregnancy, it is extremely important for women with chronic medical conditions to have a well-planned pregnancy. It is also equally important to avoid unplanned pregnancy among the women who do not intend to get pregnant, by using effective contraceptive methods. As recommended by the Preconception Care Workgroup and the Select Panel on Preconception Care, Centres for Disease Control and Prevention (CDC), optimal pre-conception care which include adequate spacing of childbirth and effective contraception are among the important elements to ensure successful maternal and fetal outcome among women with chronic medical conditions (Johnson et al., 2006).

The numbers of young women in reproductive age group who have chronic medical conditions were noted to be increasing over recent years in Malaysia and other parts of the world. The National Health and Morbidity Survey conducted in Malaysia in 2006 involving 16,440 subjects reported increasing prevalence of both hypertension and diabetes among women aged 45 years and younger (Letchuman et al., 2010; Rampal et al., 2008). Presence of surgical advancement and treatment modalities has also contributed to the longer survival of both congenital and acquired heart diseases patients as reported in the Malaysian Cardiothoracic Surgery Registry, leading to the increasing number of reproductive women with the condition (Anas et al., 2008). Similar trend were seen in other developing Asian countries and the developed world (WHO, 2011a). With the increasing number of younger women with chronic medical conditions who would be facing greater reproductive risk as compared to healthy women, it would be important to have more information and better understanding on their reproductive health. One important aspect of it is regarding the prevalence and pattern of contraceptive use, especially among women who do not intend to conceive in the near future.

According to a national survey on reproductive health and the use of contraceptive which was conducted in 2004, only 34.4% of married Malaysian women practiced modern contraceptive methods (National Population and Family Development Board Malaysia, 2004). In the same survey, contraceptive prevalence rate (CPR) was reported to be 51.8%, which combined the prevalence of both modern and traditional contraceptive practices. This is quite low as compared to other Asian countries. In Thailand, the CPR among reproductive women based on a national survey in 2004 was reported as 79.2% (Ministry of Health Thailand, 2003). High percentage of contraceptive use was also noted among women in Vietnam where the average CPR reported between the year 2000 to 2008 was 79.0% (World Health Organization, 2011b). According to the Indonesian Demographic and Health Survey in 2002 and 2003, about 56.4% of married Indonesian women used modern contraceptive methods (Ministry of Health Republic of Indonesia, 2003). The low prevalence of contraceptive use among Malaysian women showed that there is a significantly high unmet needs for contraception, which may expose the women to the risk of unplanned pregnancy.

Limited evidences were available on contraceptive use among women with higher reproductive risk in Malaysia, such as women with chronic medical condition. A report by the Ministry of Health Malaysia in 2008 regarding national analysis of confidential enquiry into maternal deaths (CEMD) showed between 60% to 70% of pregnant mothers who died in Malaysia between 2001 to 2005 never practice any form of contraception (Ministry of Health Malaysia, 2008). Another piece of information was from a pilot project which aimed to improve the reproductive outcome of women with history of high risk pregnancy. This project was conducted in three rural clinics in Malaysia from 2004 to 2006. The result showed that about three quarter of the women used modern contraception for at least 2 years after their delivery (Rosliza & Majdah, 2010). However, the pilot project only reported cumulative prevalence of contraceptive use and did not specify contraceptive use based on the different pregnancy risk such as presence of chronic medical condition. Therefore, actual prevalence of use among women with chronic medical condition could be much lower than the cumulative prevalence reported.

Given the scarcity of knowledge in contraceptive use among women with chronic medical condition, this study was conducted to get an overview of contraceptive use in terms of prevalence and methods used. In addition to that, this study also examined the factors associated with contraceptive non-use among these women, which will provide useful information for health intervention and promotion in the future. This study only focused on women with chronic medical condition who do not intend to conceive in the next one year, as these women have clear indication for contraceptive use to prevent the unplanned pregnancy.

## 2. Methods

### 2.1 Study Sample

This study used data from a family planning survey which was conducted in the specialist clinic of health facilities in a southern state of Malaysia. There were three hospitals with medical specialist outpatient clinic and 8 health clinics with resident family physicians offering outpatient primary care in the state. The hospitals were located in the urban area while the health clinics were serving the sub-urban or rural communities. In this study, one hospital and two health clinics were selected randomly to represent the urban & sub-urban/rural communities respectively. All eligible women who attended the selected clinics between January and August 2011 were invited to participate in the survey. Participants were selected among married women of reproductive age group between 18 to 50 years who has been diagnosed as having at least one chronic medical condition including diabetes, hypertension, heart disease and connective tissue diseases. The connective tissue diseases were systemic lupus erythematosus, scleroderma, rheumatoid arthritis and mixed connective tissue disease. Women who were menopaused, have had hysterectomy or bilateral tubal ligation, or whose husbands have had vasectomy were excluded from the survey. Women were eligible for the present analyses if they do not have intention to conceive in the next one year despite being sexually active. This information was captured in the questionnaire asking whether they plan to have any child in the next one year, with dichotomous answer of ‘yes’ or ‘no’. Women who answered ‘no’ were categorized as having no intention to conceive in the next one year. Therefore, these women had indication for contraceptive use and would be at risk of unplanned pregnancy if they do not use any form of contraception. In total, there were 450 women who met the inclusion criteria.

### 2.2 Definition of the Dependent Variable

The outcome variable in this study was contraceptive non-use. In the self-administered questionnaire, women were asked whether they were currently using contraceptives or not. If they answered ‘yes’, they were further asked on the type of contraceptive that they currently used. Only current users of contraceptive pills, injection, intra-uterine device, implants or condoms were considered as ‘contraceptive users’. Those who do not use any contraception or used other methods such as withdrawal, safe period, herbal medicine or other traditional practices were classified under ‘contraceptive non-users’. Withdrawal, safe period and herbal medicine were not considered as effective contraceptive methods as defined by the World Health Organisation guideline on contraceptive use (World Health Organization, 2007).

Contraceptive non-use was chosen as the dependent variable because it is important to identify the characteristics of women who were not using contraceptive. This information is useful in the process of designing any health promotion programme to encourage contraceptive use because the programme will target primarily on women who were not using contraceptive rather than current users.

### 2.3 Definition of Independent Variables

The independent variables in this analysis were reproductive history, type of health facility attended, perception towards contraceptive practice and socio-demographic characteristics. Reproductive history includes number of children and history of perinatal death. The types of health facility attended were divided into two, hospital or health clinic. The hospital represents urban health facility and the health clinic represents rural health facility. Perception towards contraception was measured by using a list of 11 statements with 5-point Likert Scale agreement options which were given 1 to 5 marks. The statements given were mainly regarding common misperceptions and taboos related to contraceptive use among Malaysian which was derived from information gathered in the National Population and Family Survey (National Population and Family Development Board Malaysia, 2004). Several examples of the statements used in the perception scale were: ‘contraceptives are not suitable for newly married couple’, ‘taking contraceptive pills will make me put on weight’, ‘using condoms will decrease sexual pleasure’ and ‘contraceptive pills will cause permanent infertility’. The total perception score was then calculated. Higher perception score indicated better perception towards contraception. To ensure internal consistency, reliability test for the perception scale were performed, and the Cronbach’s Alpha value was 0.79.

Socio-demographic variables included in the analyses were age group (18-30, 31- 40, and 41-50), education (whether they finished secondary school or not), ethnicity (Malay, or non-Malay) and whether the women was working or a housewife.

### 2.4 Statistical Analysis

Data analyses were performed using STATA Data Analysis and Statistical Software. Primary analyses involved descriptive statistics regarding contraceptive use in general and according to types of chronic medical conditions, socio-demographic background, reproductive history, knowledge level and perception on contraceptives. Independent t-test was used to compare the difference of mean perception scores between women who used and those who were not using contraceptives. Chi-square test was performed to identify association between non-use of contraceptives and the categorical variables. The level of statistical significance was set at 0.05. Finally, logistic regression analysis was conducted to identify factors associated with non-use of contraceptives among the reproductive women with chronic medical conditions.

This research has been approved both by the Medical Research Ethics Committee, Universiti Putra Malaysia with reference number of UPM/FPSK/PADS/T7-MJKEtikaPer/F01LECT(JKK)-OKT(10)07 and the Medical Research Ethics Committee, Ministry of Health Malaysia with reference number of (2) dlm. KKM/NIHSEC/ 08/0804/P10-578 dated 24 December 2010.

## 3. Results

There were 450 women who met all the inclusion criteria and included in the following analysis. General characteristics of the sample are shown in Table 1. The commonest chronic medical conditions were hypertension (54.5%) and diabetes (53.3%), followed by connective tissue diseases (11.3%) and heart disease (4.2%). The number of children that the women had ranged from one to eleven. About 8% of them did not have any child. Slightly more than half of the women were in the older age group (41-50). Majority of them were Malay, finished secondary education and received their medical care in the health clinics.

**Table 1:** Characteristics of reproductive women with chronic medical conditions ages 18-50 (n=450)

	n (%)
**Currently using family planning**	
Yes	138 (30.7)
No	312 (69.3)

Types of medical condition*	
Hypertension	245 (54.5)
Diabetes	240 (53.3)
Connective tissue diseases	51 (11.3)
Heart disease	19 (4.2)

**Reproductive history**	
** History of perinatal death**
Yes	35 (7.8)
No	415 (92.2)
**Have children**	
Yes	412 (91.6)
No	38 (8.4)
**Number of children (among those who have children, n=412)**	
1	47 (11.4)
2	81 (19.6)
3	87 (21.1)
4	89 (21.6)
5 or more	108 (26.2)

**Type of health facility attended**	
Hospital	139 (30.9)
Health clinics	311 (69.1)
**Sociodemographic variables**	
**Age**	
18-30	59 (13.1)
31-40	138 (30.7)
41-50	253 (56.2)
**Ethnicity**	
Malay	378 (84.0)
Non-Malay	72 (16.0)
**Employment status**	
Working	209 (46.4)
Housewife	241 (53.6)
**Education (Finished secondary school)**	
Yes	355 (78.9)
No	95 (21.2)

* Total percentage is more than 100% because some of the women have more than one medical condition

### 3.1 Contraceptive Practices

A total of 138 (30.7%) women reported using modern contraceptives out of the 450 women with chronic medical conditions who have indication for contraceptive use. The remaining 312 (69.3%) women reported using no contraceptive or have used non-effective methods of contraceptive such as withdrawal, safe-period, herbal medicine and other traditional practices. Higher rate of non-use was reported among the diabetics where about 71.2% of them were not using modern contraceptive. Contraceptive use was highest among women with heart disease where about three quarter reported current use of modern contraceptive. The types of contraceptive used are shown in Table 2. In general, the most popular method was oral contraceptive pills where 55 women (40%) were using it as their method of choice out of 138 women who used modern methods. This was followed by the intra-uterine device, condoms, implants and injection being the least popular. However, among women diagnosed with connective tissue disease and heart disease, the most popular method used was the condoms.

**Table 2:** Type of contraceptives used based on medical diagnosis

	All diagnosis (n=450)	Hypertension (n=245)	Diabetes(n=240)	Connective tissue disease (n=51)	Heart disease (n=19)
a.Oral contraceptive pills	55	36	32	4	2
b.Hormonal injection	6	3	2	1	0
c.Intra-uterine device	39	21	12	2	4
d.Implants	14	7	6	3	2
e.Condoms	24	18	17	6	6
f.Other contraceptive methods*	16	10	13	6	0
g.Not using any contraceptive	296	150	158	29	5

All modern contraceptives(total a+b+c+d+e)	138 (30.7%)	85 (34.7%)	69 (28.8%)	16 (31.4%)	14 (73.7%)
Contraceptive non-user (total f+g)	312 (69.3%)	160 (65.3%)	171(71.2%)	35 (68.6%)	5 (26.3%)

* withdrawal, safe-period, herbal medicine & other traditional practices

### 3.2 Factors Associated with the Non-Use of Contraceptives

Further analysis was performed to identify the factors associated with the non-use of contraceptives. Initially, univariate analysis was done to look at the association between contraceptive non-use and each independent variable. As shown in Table 3, factors which were associated with the non-use of contraceptive after the univariate analysis were: attending health clinics as compared to the hospital for their medical care (unadjusted odds ratio [OR]=2.37, 95%confidence interval [CI]: 1.52, 3.7), not having children (unadjusted OR = 3.15, 95%CI: 1.18,10.54), not finish secondary education (adjusted OR= 4.25, 95%CI: 2.15, 9.15) and being in an older age group of 41-50 year (unadjusted OR=3.03, 95%CI:1.65,5.58). Women who were of non-Malay ethnicity, working, and do not have history of perinatal death showed higher odds of not using contraceptive, however it did not reach statistical significance (chi-square test, p>0.05).

**Table 3:** Characteristics of women with chronic medical conditions who do not use contraception

	Currently not using contraception(n=312), n (%)	Unadjusted OR (95% Confidence Interval)	p value	Adjusted OR^a^ (95% Confidence Interval)
**Reproductive history**				
**History of perinatal death**				
Yes	22 (62.9)	ref	0.386*	
No	290 (69.9)	1.37 (0.61, 2.95)		
**Have children**				
Yes	279 (67.7)	ref	0.016*	ref
No	33 (86.8)	3.15 (1.18, 10.54)		4.57 (1.66, 12.57)
**Number of children**				
1	31 (66.0)	ref	0.436^	
2	54 (66.7)	0.97 (0.45, 2.08)		
3	53 (60.9)	1.24 (0.59, 2.62)		
4	68 (76.4)	0.60 (0.27, 1.31)		
5 or more	73 (67.6)	0.93 (0.45, 1.92)		

**Perception score** (mean ± SD)	30.4 ± 3.4	Difference in mean score = 1.21 with 95%CI= 0.56, 1.84	0.003^#^	

**Type of health facility attended**				
Hospital	78 (56.1)	ref	<0.001*	ref
Health clinics	234 (75.2)	2.37 (1.52, 3.70)		1.75 (1.09, 2.79)

**Sociodemographic variables**				
**Age**				
20-30	32 (54.2)	ref	0.002^	ref
31-40	82 (59.4)	1.23 (0.66, 2.28)		1.27 (0.66, 2.44)
41-50	198 (78.3)	3.03 (1.65, 5.58)		2.31 (1.19, 4.48)
**Ethnicity**				
Malay	256 (67.7)	ref	0.090*	
Non-Malay	56 (77.8)	1.67 (0.90, 3.24)		
**Employment status**				
Working	146 (69.9)	ref	0.822*	
Housewife	166 (68.9)	0.96 (0.62, 1.46)		
**Education (Finished secondary school)**				
Yes	228 (64.2)	ref	<0.001*	ref
No	84 (88.4)	4.25 (2.15, 9.15)		2.87 (1.43, 5.77)

* comparison were made using chi-square test

^ comparison were made using chi-square test for trend

# comparison were made using independent sample t-test with equal variances

a Adjusted for type of health facility attended, age, having children and education. Pseudo R^2^= 0.0913

From the univariate analyses, it was noted that contraceptive non-users had lower perception score, indicating poorer perception on contraceptive as compared to contraceptive user. The difference was statistically significant (t-test, p=0.003). However, the difference in mean score was only by 1.21 point (95% CI: 1.52, 3.70) and it was too small to be clinically significant. Therefore, it was not included in the following multivariate analysis. The result of the logistic regression analysing factors associated with contraceptive non-use are shown in Table 3. The analyses was adjusted for type of health facility attended, having children, age and education. The Hosemer-Lameshow chi-square test gave a p-value of 0.955 indicating that the model fit the data adequately. Adjusted analysis showed that women who do not have any child were 4.6 times more common not to use contraception as compared to women who have children (95% CI: 1.66, 12.57). Contraceptive non-use was also more common among women who attended the health clinics as compared to women who went to seek for medical care in the hospital (adjusted OR=1.7, 95% CI: 1.09, 2.79). Adjusted analysis also showed that contraceptive non-use was more common among older women as compared to younger women. Women of between 41-50 year were about 2.3 times more common not to use contraceptive as compared to women aged 18-30 (95% CI: 1.19, 4.48). However, no significant difference in contraceptive use was noted between women age 18-30 and 31-40. Women who do not finish secondary schools were about 3 times more common not to use contraceptive as compared to their friends who completed secondary school (95% CI: 1.43, 5.77).

## 4. Discussion

From this study, it was revealed that about 70% of women who had chronic medical conditions did not use modern contraceptive methods despite being sexually active and not planning to conceive within a year. Adjusted analysis showed that contraceptive non-use were significantly associated with the type of health facility attended, having children, being in older age group and education level.

The prevalence of contraceptive use in this study was slightly lower than the national prevalence (34.4%) (National Population and Family Development Board Malaysia, 2004). However, the population involved in the two studies were different. For this present study, women who participated were older, where more than half of them aged between 41 to 50 year compared to the national survey which was participated mainly by women in 20-40 year age group. Nevertheless, low prevalence of contraceptive use indicates that there was a high unmet need of contraception among women with chronic medical conditions, which may lead them to unplanned pregnancy. Having diagnosed with hypertension, diabetes, connective tissue diseases or heart disease has already put them at a higher risk if they were to get pregnant as compared to a healthy women. Having an unplanned pregnancy will certainly add more risk and lead to higher maternal morbidity and poorer fetal outcome (Klima, 1998; Kuroki et al., 2008). This fact highlighted the importance of having a specific intervention programme to improve preconception care among women with chronic medical condition, and increase the contraceptive uptake among women who do not intend to conceive despite being sexually active to avoid unplanned pregnancy.

This study have shown that women who received their medical treatment in the health clinics were about 1.7 times more commonly not using contraceptive as compared to women who received their medical care in the hospital specialist clinic. One of the differences of these two settings was the type of health personnel attending to the patient. Clinics in hospital setting were conducted fully by medical officers and specialists while health clinics were mostly conducted either by a medical officer or paramedic. The family physicians that are available in the health clinics will mostly see complicated cases that are referred to them. This may reflect the different emphasis given by different level of health personnel in advising on family planning and contraceptive choices. Another aspect that was not looked into in this study was the possibility of lack of advice on contraceptive given by these health care providers. Previous studies have shown that quite a high percentage of women with chronic medical conditions did not use contraceptive because they did not receive any advice about it during their encounter with the medical personnel (Lakasing & Khamashta, 2001; Rogers et al., 2007). Further studies looking at the role of health providers in providing contraceptives should be conducted to have a better understanding of this issue in the different set up of Malaysian healthcare delivery system.

Another difference between these two settings was the type and level of diseases or medical conditions seen at these venues. Medical conditions seen in the hospital is obviously more complicated than those seen regularly in health clinic. The different level of seriousness in the disease may depict the level of motivation and cooperation a person will show towards his or her disease management. This could also support the findings that the percentage of contraceptive use among women with heart disease was higher as compared to other medical diagnoses.

Women in the 41 to 50 year age group were more commonly not using contraceptive as compared to the younger age group. Similar pattern was seen in the national family planning survey (National Population and Family Development Board Malaysia, 2004), as well as other studies involving women with chronic medical condition in the United States (Chuang et al., 2005; Lakasing & Khamashta, 2001). There may be several reasons why they do not keen to use contraceptives. It could be due to perceived low fertility as their age increasing or having misperception of contraindication to certain methods in the context of age (Chuang et al., 2005; Collumbien, Gerressu & Cleland, 2004). Other reasons could be due to minimal attention given to reproductive health compared to the existing medical problems by the attending health provider as the women became older, possibly due to increasing complexity of the medical problems. This could be especially true if the women were having more than one medical diagnosis which was quite a common scenario among diabetics and hypertensive patients.

This study is the first report describing contraceptive use among women with chronic medical conditions in Malaysia. It provides an initial investigation into contraceptive use among these women. However, it has several limitations. The respondents were selected only among women with chronic medical conditions who attended government health facilities. Therefore, information regarding contraceptive use among women who received treatment in the private health facilities was not captured by this study. There is a possibility of having a more affluent and educated group of women attending private health facilities which may reveal a different prevalence of contraceptive use. Due to limited resources, this study also did not examine the provider factors of contraceptive service which is an important aspect influencing its use. Finally, there is limitation in generalizability of the findings, because this study was limited to the three health facilities that participated in the family planning survey.

In conclusion, this study revealed a high percentage of contraceptive non-use among reproductive women with chronic medical conditions in a southern state of Malaysia despite their higher pregnancy risk. Several factors were noted to be significantly associated with contraceptive non-use which includes the type of health facility attended, having children, being in older age group and lower education level. The high unmet need of contraceptive among these women warrant an effective health promotion programme to encourage the uptake of contraceptives especially targeting women of older age group, low education and those who received their medical treatment at health clinics. It is important to reduce the rate of unintended pregnancy among women with chronic medical conditions towards improving pregnancy related health outcomes through preconception health optimization and planned pregnancies.
